# Development and validation of a supervised deep learning algorithm for automated whole‐slide programmed death‐ligand 1 tumour proportion score assessment in non‐small cell lung cancer

**DOI:** 10.1111/his.14571

**Published:** 2021-11-16

**Authors:** Liesbeth M Hondelink, Melek Hüyük, Pieter E Postmus, Vincent T H B M Smit, Sami Blom, Jan H von der Thüsen, Danielle Cohen

**Affiliations:** ^1^ Department of Pathology Leiden University Medical Centre Leiden The Netherlands; ^2^ Department of Pulmonology Leiden University Medical Centre Leiden The Netherlands; ^3^ Aiforia Technologies Oy Helsinki Finland; ^4^ Department of Pathology Erasmus Medical Centre Rotterdam The Netherlands

**Keywords:** artificial intelligence, computational pathology, immunotherapy, non‐small cell lung cancer, programmed death‐ligand 1

## Abstract

**Aims:**

Immunohistochemical programmed death‐ligand 1 (PD‐L1) staining to predict responsiveness to immunotherapy in patients with advanced non‐small cell lung cancer (NSCLC) has several drawbacks: a robust gold standard is lacking, and there is substantial interobserver and intraobserver variance, with up to 20% discordance around cutoff points. The aim of this study was to develop a new deep learning‐based PD‐L1 tumour proportion score (TPS) algorithm, trained and validated on a routine diagnostic dataset of digitised PD‐L1 (22C3, laboratory‐developed test)‐stained samples.

**Methods and results:**

We designed a fully supervised deep learning algorithm for whole‐slide PD‐L1 assessment, consisting of four sequential convolutional neural networks (CNNs), using aiforia create software. We included 199 whole slide images (WSIs) of ‘routine diagnostic’ histology samples from stage IV NSCLC patients, and trained the algorithm by using a training set of 60 representative cases. We validated the algorithm by comparing the algorithm TPS with the reference score in a held‐out validation set. The algorithm had similar concordance with the reference score (79%) as the pathologists had with one another (75%). The intraclass coefficient was 0.96 and Cohen’s *κ* coefficient was 0.69 for the algorithm. Around the 1% and 50% cutoff points, concordance was also similar between pathologists and the algorithm.

**Conclusions:**

We designed a new, deep learning‐based PD‐L1 TPS algorithm that is similarly able to assess PD‐L1 expression in daily routine diagnostic cases as pathologists. Successful validation on routine diagnostic WSIs and detailed visual feedback show that this algorithm meets the requirements for functioning as a ‘scoring assistant’.

## Graphical abstract

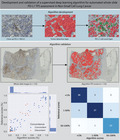

## Introduction

The 5‐year survival rate of patients with stage IV non‐small cell lung cancer (NSCLC) is poor, and this, combined with 2 million new patients annually, makes lung cancer the leading cause of cancer deaths in the world.^1^, ^2^ Immune checkpoint therapy (immunotherapy) targeting the programmed cell death protein 1/programmed death‐ligand 1 (PD‐L1) pathway^3^ has greatly improved survival for NSCLC patients.^4^, ^5^, ^6^ However, response varies greatly between NSCLC patients. Therefore, immunohistochemical PD‐L1 expression is currently used as a biomarker to select patients for immunotherapy.

**Figure 1:**
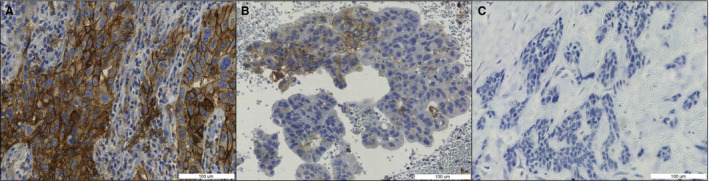
Categories of programmed death‐ligand 1 (PD‐L1) expression, measured as the tumour proportion score (TPS) (Formula 1 in Data S1). Blue staining: haematoxylin. Brown staining: PD‐L1. **A,** TPS of 50–100%. **B,** TPS of 1–49%. **C,** TPS of <1%.

Pathologists measure PD‐L1 expression by estimating the percentage of tumour cells with membranous PD‐L1 positivity [the tumour proportion score (TPS); see also Formula 1 in Data S1].^7^, ^8^ The TPS is a continuous score between 0% and 100%, and patients are further divided into three classes, i.e. TPS of <1%, TPS of 1–49%, and TPS of >50%, as outlined in Figure 1.^5^ These classes have different treatment options, provided that no targetable mutation (*EGFR*) or fusion (*ALK*; *ROS1*) is detected.^9^

Unfortunately, this PD‐L1 expression scoring system has been proven to be imperfect. The study by Cooper *et al*. showed that problematic interobserver and intraobserver discordance exists, with disagreement between pathologists in 15.8% of cases around the 1% cutoff point (*κ* coefficient: 0.68) and disagreement between pathologists in 18.1% of cases around the 50% cutoff point (*κ* coefficient: 0.58). This study points out that individual pathologists change their assessment in 8–10% of cases and that 1 h of training does not help in improving concordance.^10^ These data suggest that patients receive suboptimal treatment due to misclassification, possibly making them suffer from unnecessary side‐effects^11^, ^12^ or purposelessly increasing the already substantial costs of advanced NSCLC treatment. PD‐L1 TPS assessment could therefore benefit from computational analysis, which eradicates intraobserver variance and has the potential to eliminate some of the human factors that lead to the high rate of interobserver discordance.

Three computational PD‐L1 TPS scoring methods have been proposed in the literature so far,^13^ all of which produce high rates of concordance with the reference scores and therefore constitute a relevant proof of concept that computer‐aided PD‐L1 scoring is possible. However, the proposed algorithms all have similar limitations hampering their performance (and therefore their implementation in clinical practice) beyond the research domain. The limitations include: the use of tissue microarrays (TMAs) [making them not applicable to whole slide images (WSIs) with benign tissue backgrounds], the use of trial material instead of clinical material (resulting in only easy‐to‐score material being present in the validation set), a limited number of observers for the ground truth score, a lack of precise predictions (undermining the algorithm’s explainability for clinicians), requiring manual annotations for each scoring area (resulting in a very labour‐intensive process and potential sampling error), and being thresholding‐dependent (making them not transferable to a clinical setting, in which staining intensity varies over time). For all of these algorithms, the question is whether they are reliable in a clinical setting. Detailed descriptions of the different study setups and potential limitations are included in Table S1.^14^, ^15^, ^16^

## Materials and methods

To summarise, the perfect PD‐L1 algorithm does not yet exist. A good, practically usable PD‐L1 algorithm should be trained and validated on WSIs that originate from routine diagnostics. In order to correctly assess the PD‐L1 TPS within the wide variety of tissue contexts from which NSCLC stage IV biopsies originate (benign bronchial epithelium, lymph nodes, adrenal gland, bone and cartilage, skin, liver, kidney, etc.), and also to correctly neglect positive immune cells such as macrophages, a deep learning‐based approach is required. Additionally, because of the high interobserver variance in PD‐L1 scoring that the algorithm is intended to overcome, the reference scores should be acquired from multiple observers rather than just one. Finally, the algorithm should provide visual feedback at a microscopic level, in order to make algorithm scores interpretable for pathologists and pulmonologists. These criteria are outlined in Table 1. In this article, we therefore present the first fully supervised deep learning PD‐L1 TPS algorithm, based on a cohort from routine diagnostics with robust reference scores generated by three experienced thoracic pathologists.

**Table 1:** Criteria for algorithm applicability to clinical diagnostics

Study setup feature	Criterion for applicability to clinical diagnostics
Case selection	Routine diagnostic cases, including ‘difficult’ features, e.g. metastasis tissue background or artefacts
Ground truth	As robust as possible: multiple expert observers or response data
Validation	Validation at the whole slide level
Algorithm feedback	Easily interpretable, detailed visual feedback

### CASE SELECTION

One hundred and ninety‐nine consecutive NSCLC specimens from routine diagnostics at the Leiden University Medical Centre, for which PD‐L1 staining had been performed for routine diagnostics and the TPS was registered in the pathology report, were included. Cases were excluded if the patient (at the time of the biopsy) did not give permission for the use of leftover tissue for research purposes, if a small‐cell or neuroendocrine morphology was described, or if the biopsy contained <100 tumour cells.

The samples originated from both in‐house and referral cases. Three cytology cases with large tumour islands resembling histology specimens were included; all other cytology cases (including all endobronchial ultrasound‐guided transbronchial needle aspiration specimens) were excluded. Patients with a second primary NSCLC on which PD‐L1 staining had also been performed were included twice (both tumours once). Both metastasis biopsies and primary tumours were included. All samples were irreversibly anonymised after inclusion, by use of a unique four‐digit random number.

### PD‐L1 STAINING METHODS

Slides were stained for routine diagnostics, over a period of several years. Formalin‐fixed paraffin‐embedded blocks were cut into 3‐µm sections with a Leica RM2255 Automated Microtome (Leica Biosystems B.V., Amsterdam, the Netherlands). Sections were placed on microscope slides and dried at either 60°C for 30 min to 16 h, or at 37°C for 72 h. After being dried, the slides were deparaffinised, and antigen retrieval was performed in citrate buffer (Target Retrieval Solution, pH 6) for 40 min. Immunohistochemistry (IHC) was performed according to a laboratory‐developed test protocol. Slides were stained with the Dako Omnis immunostainer and Dako EnVision Flex+ reagents and 1:20 dilution of PD‐L1 clone 22C3 (Dako Omnis, Dako Agilent Technologies, Leuven, Belgium). The IHC slides were then counterstained with haematoxylin, and coverslips were applied. Tonsil and placental tissue were used as positive controls for PD‐L1 expression.

### SCORING

All of the 199 included samples were independently scored (TPS; Data S1, Formula 1) by three trained pulmonary pathologists (D.C., J.T., and V.S.). The pathologists were blinded to each other’s scores. The continuous TPS was divided into three categories (<1%, 1–49%, and 50–100%) for part of the analyses. The level of concordance between the pathologists was calculated by making 597 pairwise comparisons from the 199 scored cases. If the paired pathologists scored in the same category (<1%, 1–49%, and 50–100%), the case was considered to be ‘concordant’. For comparison with algorithm performance, we calculated the mean of the three pathologists’ continuous TPSs and used that as the reference score for the algorithm (Formula 2 in Data S1).

### SCANNING

We anonymised glass slides before scanning, by generating random barcodes for each slide. Digital WSIs were acquired with Nanozoomer 2.0‐HT (Hamamatsu Photonics, Hamamatsu City, Japan) scanners at a resolution of 0.23 µm/pixel. The WSI metadata did not contain any personal data. WSIs were uploaded to the Aiforia Hub platform (Aiforia Technologies, Helsinki, Finland) as.ndpi files without additional processing.

### TRAINING AND VALIDATION SET

A training set of 60 samples was selected from the 199 included cases. In the training set, there was variance in tumour type, biopsy site, tissue size (tumour resection or small core needle biopsy), and the TPS. We included extra lymph node biopsies and squamous cell carcinomas in the training set, because only a handful of these cases were included in the training set when we selected randomly. All remaining samples were included in the validation set, which resulted in a held‐out validation set of 139 cases.

### ALGORITHM SETUP

**Figure 2:**
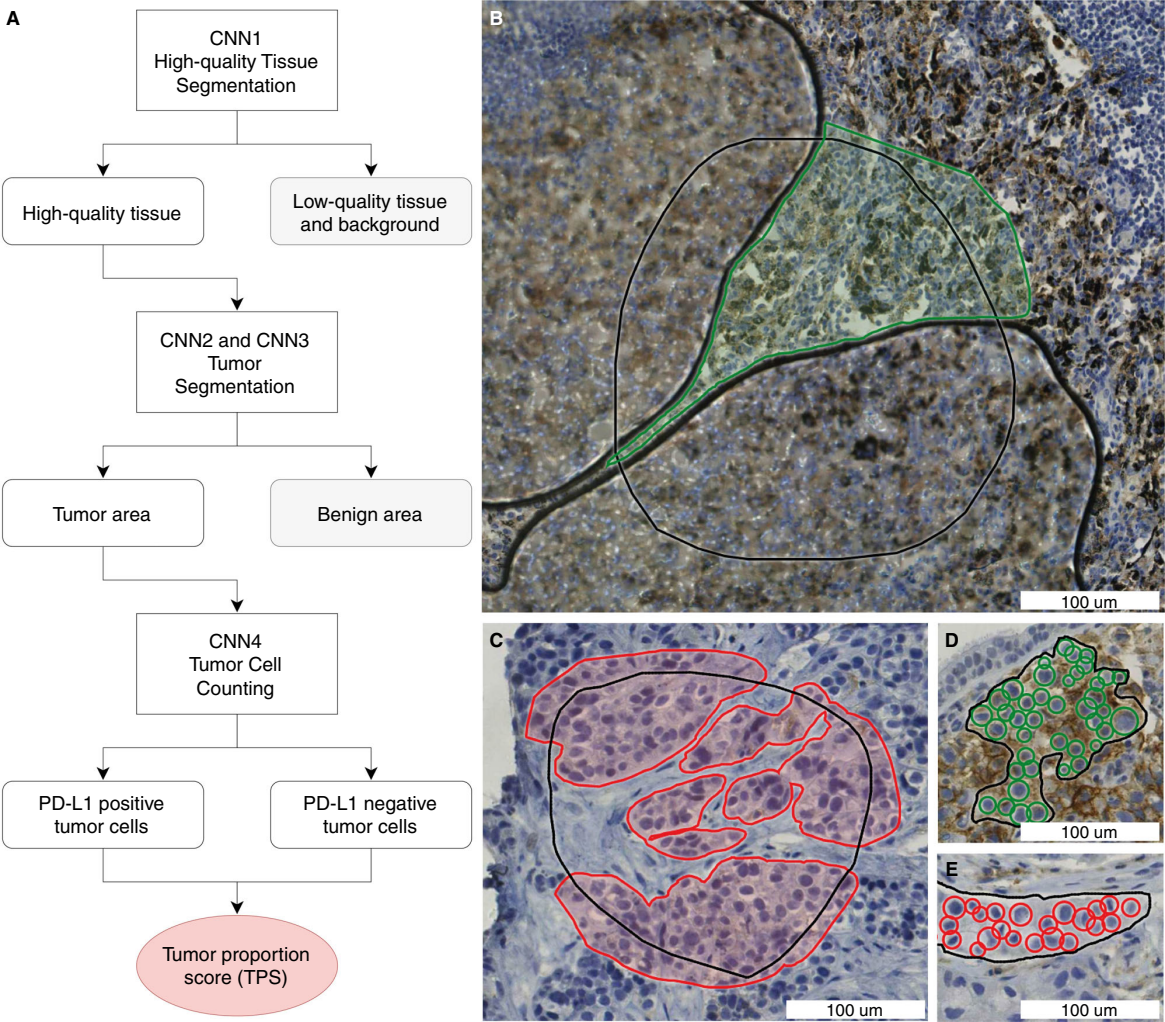
Algorithm setup and annotations. **A,** Schematic algorithm setup with four convolutional neural networks (CNNs) to calculate the programmed death‐ligand 1 (PD‐L1) tumour proportion score (TPS). **B,** Annotations for high‐quality tissue segmentation (CNN1). Green annotated: high‐quality tissue that is in focus and does not contain artefacts. Black: annotated region of interest (ROI), and non‐annotated area within the ROI: the tissue is of low quality in this example, because of air bubbles. **C,** Annotations for tumour segmentation (CNN2 and CNN3). Red annotated: tumour. Black: annotated ROI, and non‐annotated area within the ROI: non‐neoplastic tissue. **D,E,** Annotations for tumour cell counting (CNN4). Green: annotated PD‐L1‐positive nuclei. Red: annotated PD‐L1‐negative nuclei. Black: annotated ROI. The TPS can be calculated from the number of PD‐L1‐positive tumour cells and the number of PD‐L1‐negative tumour cells (Formula 1 in Data S1). All annotations were placed in the training set (*n* = 60), which was withheld from validation.

The algorithm consists of four separate convolutional neural networks (CNNs) (Figure 2) and is programmed in C++. The first three CNNs are binary semantic segmentation models. The first CNN segments high‐quality tissue versus background or low‐quality tissue. The class ‘low‐quality tissue’ includes white background, out‐of‐focus tissue, folding artefacts, air bubbles, glass edges, and other tissue that is of too low quality to be used for scoring. As the PD‐L1 TPS score must score only tumour cells and neglect immune cells, such as macrophages, the second and third CNNs both segment neoplastic tissue versus all other high‐quality tissues. Both CNNs use precisely the same annotations, but the second CNN utilises a larger tile size (200 µm)—which results in coarse segmentation—whereas the third model uses smaller tiles (50 µm) and is used to refine the predictions of the second CNN. This method of refining segmentation predictions enabled more precise prediction of neoplastic cells and islets, and has not been described before for pathology image analysis. The fourth CNN is an object detection model with two classes: PD‐L1‐positive cells and PD‐L1‐negative cells. Each CNN is used only within the segmented area of the previous CNN, which, for example, results in the ignoring of PD‐L1‐positive and PD‐L1‐negative immune cells outside of the neoplastic areas. The four‐CNN setup was chosen in order to mimic human scoring, and to enhance explainability to clinicians and patients.

### ANNOTATIONS

**Figure 3:**
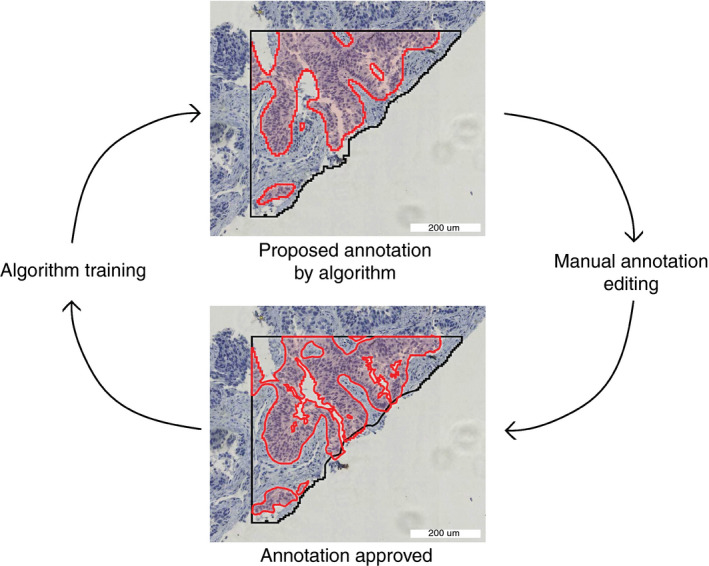
Human in the artificial intelligence loop (HAIL) annotation method. Red: neoplastic tissue. Black: region of interest. The preliminary algorithm proposes annotations, which can be approved, edited or rejected by the annotator. This process speeds up annotating and enables screening for ‘difficult’ features early in the algorithm development process. In each HAIL cycle, multiple annotations are proposed, edited, and accepted. [Colour figure can be viewed at wileyonlinelibrary.com]

All annotations were placed by the same trained annotator (L.H.), under the supervision of thoracic pathologists D.C. and J.T., in regions of interest (ROIs) in the training set (60 WSIs). Examples of annotations are shown in Figure 2 and Figure S1. In order to speed up the last part of the annotation process, we used an adaptation of the human in the artificial intelligence (AI) loop (HAIL) method, as outlined in Figure 3.^17^ In this method, the preliminary AI model proposes annotations that can be approved, edited or rejected by the annotator. This process substantially speeds up annotating, as previously described in the literature,^17^ and enables screening for ‘difficult’ features early in the algorithm development process. All annotations were placed in the training set (*n* = 60), which was not used for validation.

### ALGORITHM TRAINING AND VALIDATION

Algorithm training and validation were performed with aiforia v4.6, as previously published.^18^, ^19^ The error against annotated training data was used as an evaluation metric for each CNN separately. The loss function for semantic segmentation networks was multiclass logistic regression. For the object detection network, a custom‐built loss function was used within the aiforia panel. For each CNN, augmented tiles (the augmentation settings are outlined in Figure S2) were used: CNN1, 8 052 800 tiles; CNN2, 5 860 000 tiles; CNN3, 6 472 800 tiles; and CNN4, 58 201 600 tiles.

For validation, the algorithm was applied to all WSIs in the validation set. The algorithm TPS for each WSI was acquired and compared with the whole‐slide reference score from the pathologists. Cases were considered to be ‘concordant’ when the algorithm score and the reference score were in the same category: TPS of <1%, 1–49%, or ≥50%. Cases were considered to be ‘not scorable’ when the algorithm detected <100 neoplastic cells in the WSI. Cases were considered to be either ‘around the 1% cutoff point’ (reference score of <25%) or ‘around the 50% cutoff point’ (reference score of ≥25%).

## Results

One hundred and ninety‐nine NSCLC histology cases were included in the study. We compared our algorithm‐derived PD‐L1 TPS (algorithm score) with the mean of three scores of specialised pathologists (reference score).

### PATIENTS AND CASES

The characteristics of the training and validation set are shown in Table 2. The two groups are slightly different, which is a result of enriching the training set for lymph node biopsies and squamous cell carcinomas, as only a handful of those cases were included in the training set by random selection.

**Table 2:** Case characteristics

Characteristic	Training set (*N* = 60)	Validation set (*N* = 139)	*P*‐value
Age (years) (range)	69 (45–86)	68 (48–90)	0.7*
Sex, *n* (%)			1.0^†^
Male	35 (58)	81 (58)	
Female	25 (42)	58 (42)	
Tumour type, *n* (%)			0.03^‡^
Adenocarcinoma	44 (73)	117 (84)	
Squamous cell carcinoma	16 (27)	18 (13)	
Adenosquamous carcinoma	0	4 (3)	
Biopsy site, *n* (%)			0.01^‡^
Lung	30 (50)	89 (64)	
Lymph node	14 (23)	11 (8)	
Distant metastasis	16 (27)	39 (28)	
PD‐L1 in report, *n* (%)			0.53‡
Negative (<1%)	28 (47)	69 (50)	
Low positive (1–49%)	20 (33)	36 (26)	
High positive (50–100%)	12 (20)	34 (24)	

PD‐L1, programmed death‐ligand 1.

Significant difference are due to enriching the training set for lymph node biopsies and squamous cell morphology, as only a few of those were included when we selected the training set randomly.

* Unpaired *t*‐test.

^†^ Fisher’s exact test.

^‡^ Chi‐squared test.

### INTEROBSERVER VARIABILITY BETWEEN PATHOLOGISTS

**Figure 4:**
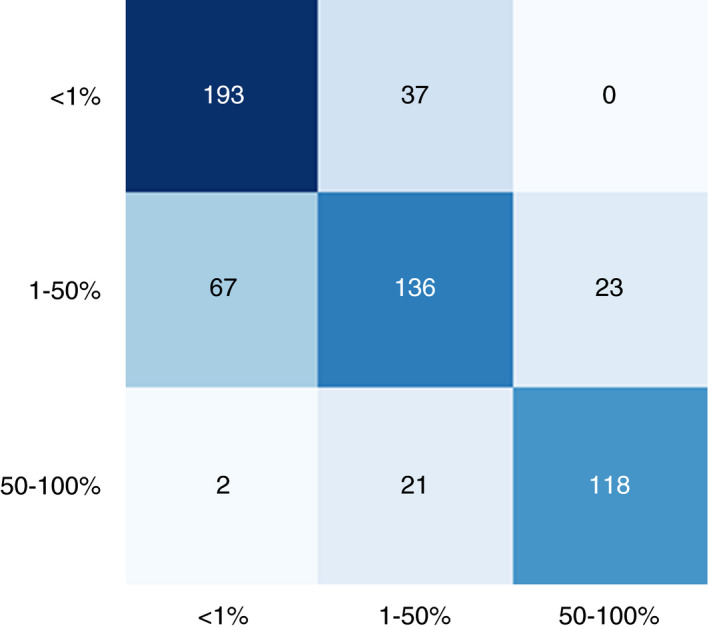
Confusion matrix for interobserver variance between pathologists. The confusion matrix is based on three observers and 199 cases, constituting 597 pairwise comparisons. One of the paired observers is plotted on the *x*‐axis and the other observer is plotted on the *y*‐axis. [Colour figure can be viewed at wileyonlinelibrary.com]

The three pathologists were in complete agreement in 124 of 199 cases (62%). In pairwise comparisons (*n* = 597; Figure 4), the overall concordance between any two pathologists was 75%. Around the 1% cutoff (136 cases), all three pathologists agreed in 83 cases (61%). There were 408 pairwise comparisons around 1%, resulting in an overall concordance of 74%. Around the 50% cutoff (63 cases), all three pathologists agreed in 41 cases (65%). Between any two pathologists in the 189 pairwise comparisons around 50%, the concordance was 77%. The Fleiss *κ* coefficient was 0.61 overall (substantial agreement; 95% confidence interval 0.612–0.616). The mean absolute difference between the pathologists’ assessments was 8%. These data are similar to the concordance rates described in the literature.^10^, ^20^, ^21^, ^22^, ^23^, ^24^, ^25^, ^26^

### ALGORITHM TRAINING AND METRICS

We trained the four CNNs separately. For each CNN, the training settings and output (tile size, amount of training data annotated, epochs trained, and error against training data) are summarised in Table 3. Error against training data was calculated with Formula 1 for semantic CNNs (CNN1, CNN2, and CNN3), and with Formula 2 for object detection CNN (CNN4). We used an early stopping mechanism, which ended the training after ⁓18 h when there was no progress in the loss function output over a set amount of epochs.

**Table 3:** Training parameters per convolutional neural network (CNN)

CNN	Tile size (µm)	Resolution ()µm/pixel	Annotated data	No. of CNN layers	Epochs trained	Error against training data (%)
CNN1: High‐quality tissue	50	1.61	517 mm^2^	8	5033	0.12
CNN2: Neoplastic tissue (coarse)	200	1.57	960 mm^2^	12	14 650	0.49
CNN3: Neoplastic tissue (refinement)	50	0.39	960 mm^2^	12	16 182	0.15
CNN4: Cell detection	86	0.44	5159 objects	6	18188	9.1

Training parameters for each CNN included tile size, resolution, the amount of annotated training data, the number of convolutional layers per CNN, epochs trained, and error against the training data. Error formulas are provided in Formulas 1 and 2.

Formula 1: Error formula for segmentation CNNs (CNN1, CNN2, and CNN3).

Error = [false‐positive area (mm^2^) + false‐negative area (mm^2^)]/ROI area total (mm^2^)

Formula 2: Error formula for the object detection CNN (CNN4).

Error = true‐positive area (mm^2^)/[true‐positive area (mm^2^) + false‐positive area (mm^2^) + false‐negative area (mm^2^)]

### ALGORITHM VALIDATION

**Figure 5:**
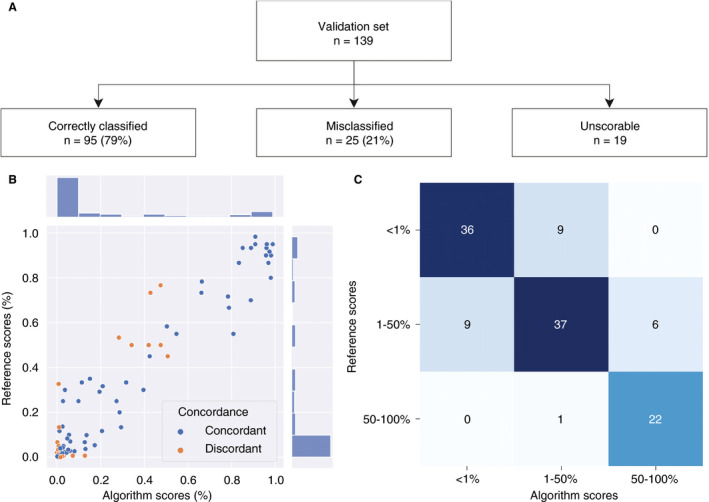
Algorithm validation set results. **A,** Flowchart for the validation process. **B,** Scatterplot for the mean of the three pathologists’ continuous tumour proportion scores (TPSs): the reference score (Formula 2 in Data S1) is on the *y*‐axis, and the continuous algorithm score is on the *x*‐axis. **C,** Confusion matrix for categorical TPSs (<1%, 1–49%, and 50–100%): the reference score categories are on the *y*‐axis, and the algorithm score categories are on the *x*‐axis. [Colour figure can be viewed at wileyonlinelibrary.com]

In the validation set, as outlined in Figure 5, the concordance between the reference score and the algorithm score was 79% overall, whereas any two pathologists agreed with each other in only 75% of the cases. The algorithm concordance was also 79% around the 1% and 50% cutoff points, whereas any two pathologists agreed with each other in 74% and 77% of the cases around these cutoff points. The average difference between any two pathologists was 8%, and the average difference between the algorithm score and the reference score was 5%, which is significantly lower (*P* = 0.01, unpaired *t*‐test). The intraclass coefficient (with a consistency definition) was 0.96 [95% confidence interval (CI) 0.94–0.97], when the continuous algorithm score was compared with the continuous reference score. The algorithm identified 39 359 neoplastic cells per slide on average (range, 188–749 558 cells). Cohen’s *κ* coefficient for the algorithm was 0.68. This is similar to the Fleiss *κ* coefficient calculated for the pathologists (0.61).

Nineteen cases were registered as ‘unscorable’ by the algorithm. In 11 cases, this was due to poor scanning quality and the WSI being out of focus (partly or completely). In five cases, there were severe artefacts, which had not been included in the training set and made the WSI difficult to score for the algorithm (Figure S3A) In both of the two remaining slides, the tumour was strongly discohesive, falling apart in such small parts that it resembled cytology, which was not included in the training set. In these cases, the algorithm did not correctly identify all of the tumour cells and counted <100 tumour cells (Figure S3B). One hundred and twenty cases remained for algorithm validation.

For cases scored <0.5% by the algorithm (*n* = 32), the concordance with the reference score was 94%. For cases scored >60% by the algorithm (*n* = 20), the concordance with the reference score was 100%. The cases with scores of <0.5% and >60% constituted 43% of the validation set (*n* = 52). Examples of algorithm applicability for both the ‘difficult’ cases (TPS of 0.5–60%) and the ‘easy’ cases are provided in Figure 7 and Figures [Link], [Link], [Link].

### EXPLAINING DISCORDANCE

**Figure 6:**
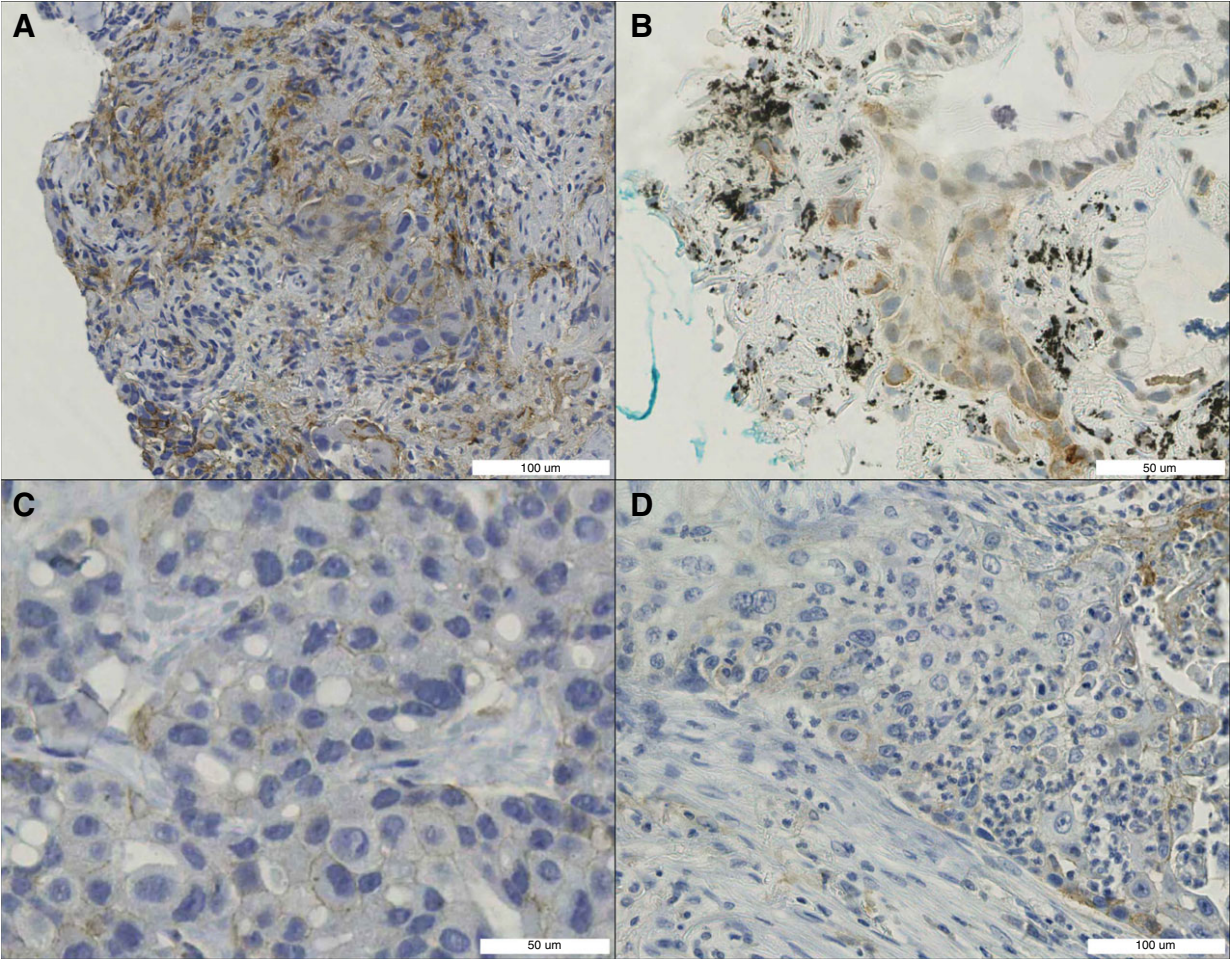
Difficult‐to‐score features. **A,** Neoplastic tissue surrounded by benign programmed death‐ligand 1 (PD‐L1)‐positive cells. **B,** PD‐L1 staining in neoplastic cells: partly nuclear, partly cytoplasmic, and partly membranous (anthracosis and ink). **C,** Low‐intensity PD‐L1 staining. **D,** PD‐L1‐positive immune cells infiltrating neoplastic tissue. [Colour figure can be viewed at wileyonlinelibrary.com]

On closer examination of the cases that were misclassified by the algorithm (orange dots; Figure 5B), it is clear that, in 20 of 25 misclassified cases (80%), the pathologists were also in disagreement, meaning that one of the pathologists scored the case in a different treatment category. This occurred significantly more frequently than in the cases that were correctly classified by the algorithm (27%, *P* = 0.000003, Fisher’s exact test), which suggests that these cases were more difficult to score for both human and machine. Common features in the misclassified cases included the following:

1. The reference score was close to the 1% or 50% cutoff point.
2. Neoplastic tissue was surrounded or infiltrated by PD‐L1‐positive immune cells (Figure 6A,D.)
3. Neoplastic cells stained for PD‐L1, but the staining was non‐membranous (Figure 6B).
4. Neoplastic cells stained for PD‐L1, but the entire membrane did not stain positively (incomplete staining) (Figure 6C).
5. Neoplastic cells stained for PD‐L1, but with low intensity (Figure 6C).
6. There were severe artefacts, including anthracosis, folds, ink, degeneration, preservation‐related issues, and scanning‐related issues (Figure 6B).
7. A small number (<250) of neoplastic cells were available for scoring.

The misclassified cases were not significantly different from the correctly classified cases with regard to tumour type (*P* = 0.5, chi‐squared test), biopsy site (*P* = 0.4, chi‐squared test), or PD‐L1 TPS category (*P* = 1.0, chi‐squared test).

### VISUAL ALGORITHM FEEDBACK

**Figure 7:**
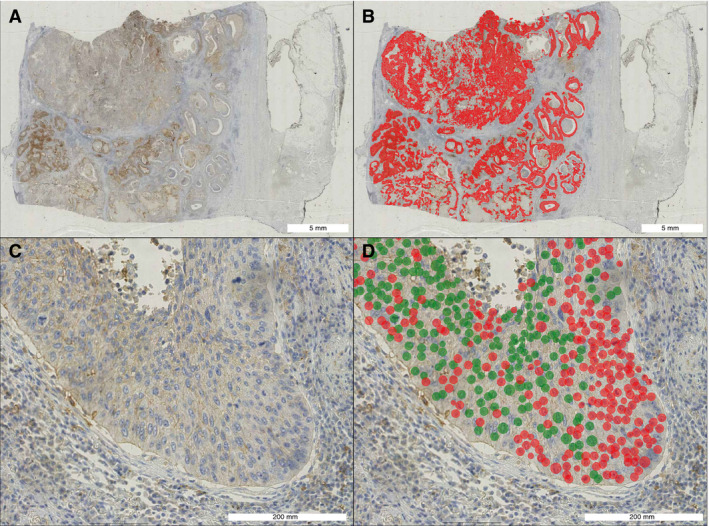
Case example algorithm scoring of a ‘difficult’ case close to the 50% cutoff. **A,** A programmed death‐ligand 1 (PD‐L1)‐stained lobectomy slide overview of a squamous cell carcinoma. **B,** Prediction from convolutional neural network (CNN) 3 (neoplastic area segmentation). Red: neoplastic tissue. **C,** Representative close‐up. **D,** Prediction from CNN4 (cell detection). Red: PD‐L1‐negative cell. Green: PD‐L1‐positive cell. In total, the algorithm counted 98 235 PD‐L1‐positive cells and 118 604 PD‐L1‐negative cells in this whole slide image, resulting in a tumour proportion score of 45.3%. The pathologists scored this case at 30%, 60%, and 45%, respectively. The reference score was therefore 45%. [Colour figure can be viewed at wileyonlinelibrary.com]

The algorithm provides detailed visual feedback of predictions, at both the whole slide level and the microscopic level. The cell counting aspect of the algorithm enables exact approximation of the TPS, whereas, obviously, pathologists can only give a rough estimate. A case example is shown in Figure 7. Additional case examples are shown in Figures [Link], [Link], [Link].

### SEGMENTATION REFINEMENT

**Figure 8:**
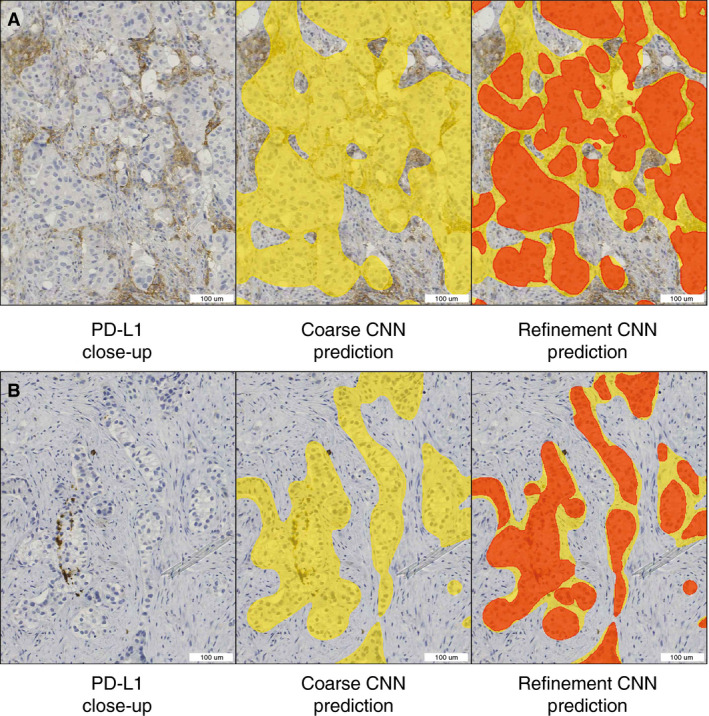
Segmentation refinement examples. Left: programmed death‐ligand 1 (PD‐L1)‐stained tissue. Middle: overlay predictions (yellow) from the first neoplastic segmentation convolutional neural network (CNN) (coarse CNN). Right: overlay predictions from the first (yellow) and second (red) neoplastic segmentation CNNs (refinement CNNs). In case A (negative tumour cells with closely associated positive immune cells), use of only the coarse CNN would have resulted in falsely counting more PD‐L1‐positive cells, and potentially a higher tumour proportion score (TPS) (false‐positive). In case B (negative cells in negative stroma), using only the coarse CNN would have resulted in falsely counting more PD‐L1‐negative cells, and potentially a lower TPS (false‐negative). [Colour figure can be viewed at wileyonlinelibrary.com]

Our algorithm utilises two sequential segmentation CNNs for neoplastic tissue detection. The first CNN (coarse CNN) has a tile size of 200 µm, whereas the subsequent CNN (refinement CNN) has a tile size of 50 µm. An example of this setup is outlined in Figure 8. Adding the refinement CNN reduces the error against the training data from 0.49% to 0.15%, which constitutes a 3.3‐fold decrease (Table 3). This approach therefore improves the predictions and decreases the required amount of annotations, as both segmentation CNNs utilise the same set of annotations. The added benefit of this approach was especially clear in cases with small patches of neoplastic tissue, as shown in Figure 8.

## Discussion

The PD‐L1 TPS is an established biomarker, with direct treatment consequences for late‐stage NSCLC patients. However, PD‐L1 as a biomarker for response to immunotherapy has several drawbacks, the most important being the high interobserver and intraobserver variance around rigid cutoff points (at 1% and 50%), and the fact that negative patients may also respond (and vice versa). Although a more definitive solution for a more accurate prediction of response to immunotherapy is still a subject of research, some of the human factors leading to high interobserver and intraobserver variance may be solved by the use of computational PD‐L1 scoring. Several attempts have been made to create PD‐L1 scoring algorithms, but all have specific limitations that hamper robust translation into clinical practice.

We therefore developed and validated a fully supervised deep learning algorithm for computational PD‐L1 scoring, which gives scores concordant with the reference score in 79% of cases, whereas any two pathologists agree with each other in 75% of cases. Cohen’s *κ* coefficient for the algorithm is 0.68 and the intraclass coefficient is 0.96; respectively, these constitute ‘substantial’ to ‘almost perfect’ agreement, and are close to the agreement rates between the three experienced thoracic pathologists in this study.

An additional strength of our algorithm is that it provides detailed visual whole‐slide predictions at a microscopic level, owing to the fully supervised setup of the model. This feedback increases interpretability and explainability, which is an important criterion for algorithms that will be used by pathologists in a clinical setting.

We believe that—in order to be of value in daily clinical practice—any algorithm should be designed with cases derived from routine diagnostic WSIs, as opposed to ‘perfect’ trial material^14^, ^15^ or TMAs.^16^ Our algorithm is trained and validated on routine diagnostic whole slide histological material, including a wide range of metastatic sites and tissue artefacts. Because of its deep learning‐based nature, the algorithm performs well in the highly heterogeneous tissue backgrounds in WSIs (artefacts, lymph nodes, bronchial epithelium, adrenal gland, skin, brain, bone, kidney, etc.), which requires extensive annotations and is not easily achieved with simpler machine learning approaches.^16^ For validation, algorithm scores were compared with the scores of multiple observers, which is essential because the reference score needs to be as robust as possible. It must be noted that our *κ* coefficient for pathologist agreement is relatively low as compared with those in some PD‐L1 interobserver studies using trial material or TMAs,^14^, ^15^ but is in line with those in other studies with similar broad inclusion criteria.^10^

Given the described accuracy and clinical applicability of our model, one may think of two different areas of usage: (i) PD‐L1 scoring in a (case‐by‐case) diagnostic setting; and (ii) PD‐L1 scoring of trial material and/or large series in a research environment. In a diagnostic setting, we see this algorithm as a potential ‘scoring assistant’ or second‐opinion tool, aiding and saving time for pathologists, especially in difficult cases. In a situation in which scoring of large series or trial material is required, this algorithm may stand alone in the scoring of ‘easy’ cases with <0.5% or >60% PD‐L1 positivity, as the algorithm reaches an accuracy of 96%. A pathologist could then focus on the subset of difficult cases with PD‐L1 scores between 0.5% and 60%. A second observer pathologist may be replaced by our algorithm. Overall, our PD‐L1 algorithm will function mostly as a scoring assistant or second observer, thereby saving time and human effort, while remaining equally accurate.

Although the implementation of this and other algorithms in daily clinical practice is imminent, the applicability of this algorithm is likely to be hampered by domain divergence (different scanners, different antibodies, different stainers, etc.). When this algorithm is used in a new laboratory, or when laboratory circumstances change, ‘domain adaptation’ (adapting the algorithm to the same task but in a new dataset) is required.

The difficulty of the domain adaption process and the choice of a method of adapting is heavily dependent on the domain relatedness (or the measure of domain divergence), which is a subject of ongoing research in the field of computer vision. Domain adaptation can be performed in many different ways (shallow adaptation, deep supervised adaptation, adversarial adaptation, semisupervised adaptation, domain matching, etc.).^27^, ^28^

Histopathology articles describing the process of domain adaptation in computational pathology are mostly lacking. We consider this to be a potential drawback. Clear guidelines for ‘domain adaptation’ and ‘post‐implementation monitoring’ will need to be established in the near future. This issue will therefore be the subject of future research, in which we will use this PD‐L1 algorithm for a nationwide PD‐L1 domain adaptation study.

Another future research challenge for the field of PD‐L1 assessment and digital pathology is its application in cytology. In cytology specimens, there is substantially less tissue context, and the task of PD‐L1 TPS assessment is therefore different and perhaps more difficult. Despite these challenges, it is often necessary to use cytology material for PD‐L1 analysis in clinical practice when no histology material is available, which is the case in up to 40% of cases.^9^ Our algorithm is not applicable to, and is not easily transferrable to, cytology specimens; a separate algorithm would have to be developed for this purpose. This algorithm would need to take the different cytological backgrounds and common cell types such as mesothelial cells, macrophages and (fragments of) lymphoid tissue into account.

In conclusion, we have developed a deep learning PD‐L1 TPS algorithm that is truly applicable to daily routine whole slide specimens. State‐of‐the‐art computational techniques such as the double segmentation CNN and the HAIL annotations worked synergistically with the clinical perspective of highly experienced thoracic pathologists in this study, and resulted in the first PD‐L1 algorithm that is accurate on routine diagnostic material, in all tissue contexts, and on WSIs. In order to create smart pathology‐based deep learning algorithms that are actually meaningful for the patients and clinicians of tomorrow, a true alliance of both clinical and computational experts is crucial.

## Conflicts of interest

S. Blom is an employee of Aiforia Technologies.

## Author contributions

L.M. Hondelink: Conceptualization, Development, Validation, Formal analysis, Investigation, Data curation, Writing original draft, Writing review and editing, Visualization. M. Hüyük: Resources, Data curation, Writing review and editing. P.E. Postmus: Resources, Writing review and editing. V.T.H.B.M. Smit: Resources, Writing review and editing. S. Blom: Development, Software, Resources, Writing review and editing, Visualization. J.H. von der Thüsen: Conceptualization, Development, Investigation, Resources, Data curation, Writing review and editing, Supervision, Project administration. D. Cohen: Conceptualization, Development, Investigation, Resources, Data curation, Writing original draft, Writing review and editing, Visualization, Supervision, Project administration.

## ethics

Cases were anonymised by use of a unique and anonymous research number. Specimens were handled according to the Code for Proper Secondary Use of Human Tissue in The Netherlands (Dutch Federation of Medical Scientific Societies). This study was approved by the local Medical Ethical Committee (B20.008).

## Supporting information


**Figure S1**. Additional examples of annotations.

**Figure S2**. Training parameters and augmentation methods per CNN.

**Figure S3**. Unscorable cases.

**Figure S4**. Case example of algorithm scoring of a ‘difficult’ case between 0.5% and 60%.

**Figure S5**. Case example of algorithm scoring of an ‘easy’ case below 0.5%.

**Figure S6**. Case example of algorithm scoring of an ‘easy’ case above 60%.

**Table S1**. PD‐L1 algorithms in the literature and potential limitations.

**Data S1**. Supplementary formulas.
