# Supplementary figures and images for: Development and validation of a supervised deep learning algorithm for automated whole‐slide programmed death‐ligand 1 tumour proportion score assessment in non‐small cell lung cancer
Source: Histopathology. 2021 Nov 16;80(4):635–47. doi: 10.1111/his.14571 (PMC9299490; doi:10.1111/his.14571)

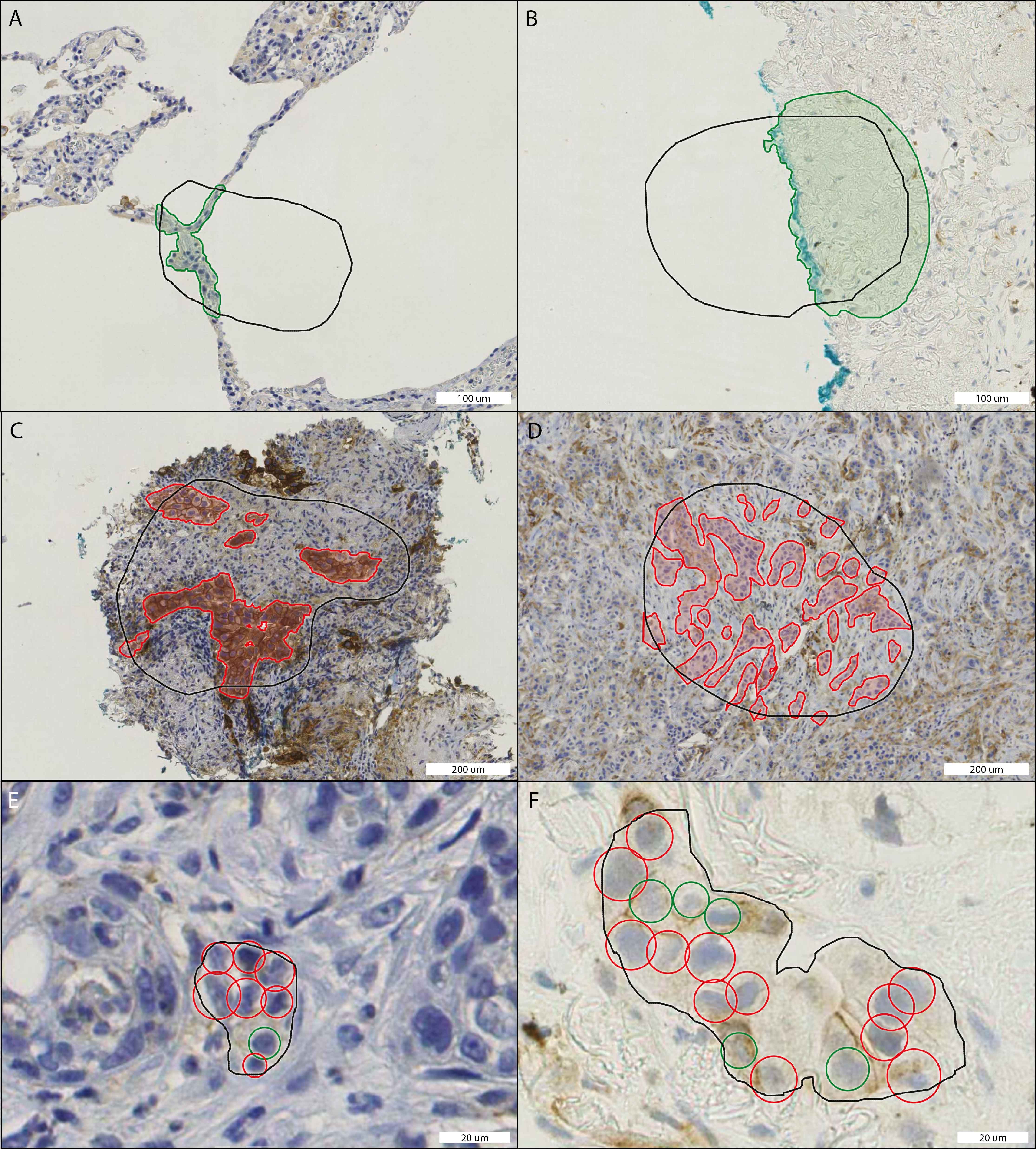

Supplement: Supplementary file 1 — Figure S1. Additional examples of annotations. [file HIS-80-635-s006.jpg]

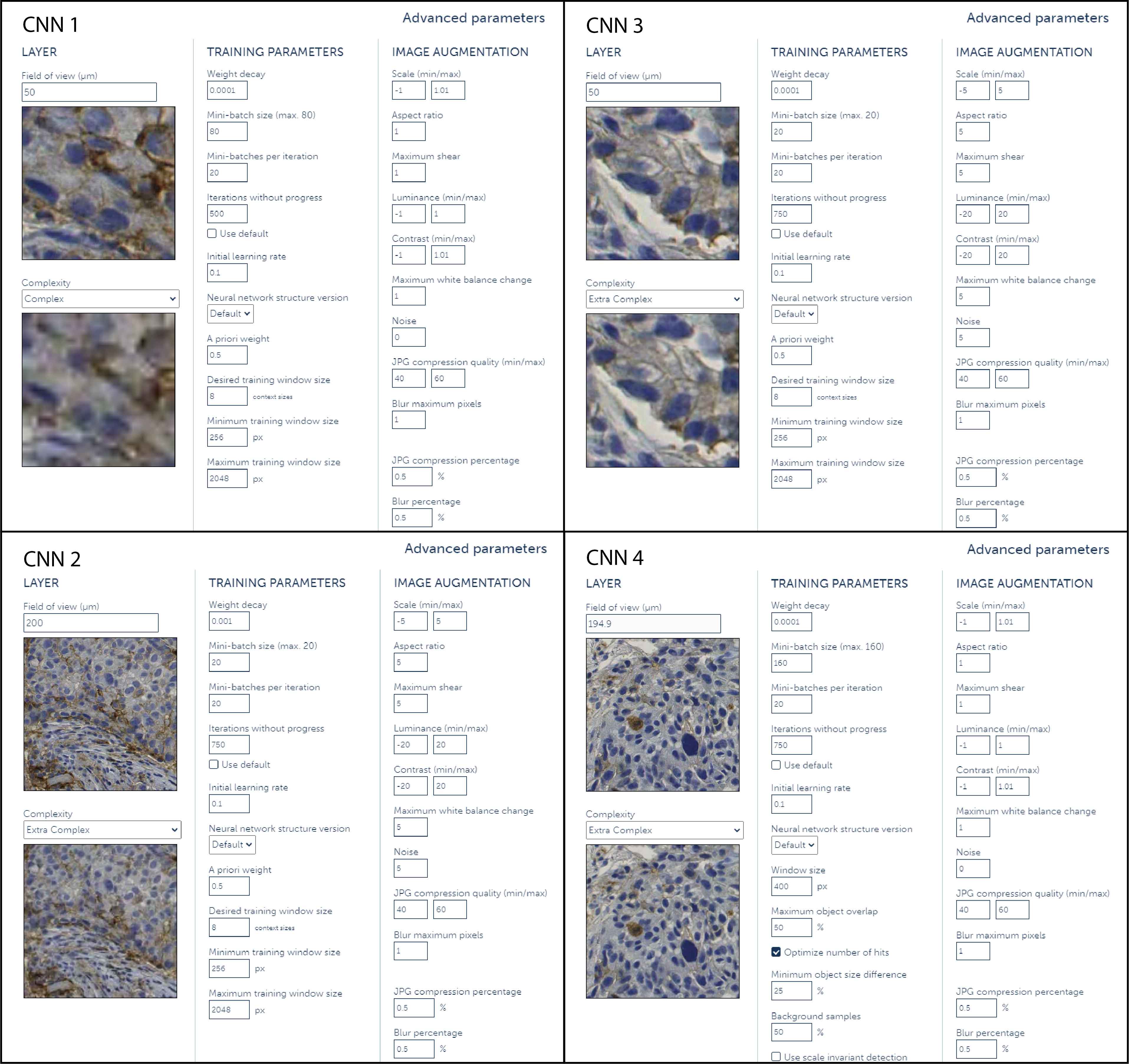

Supplement: Supplementary file 2 — Figure S2. Training parameters and augmentation methods per CNN. [file HIS-80-635-s002.jpg]

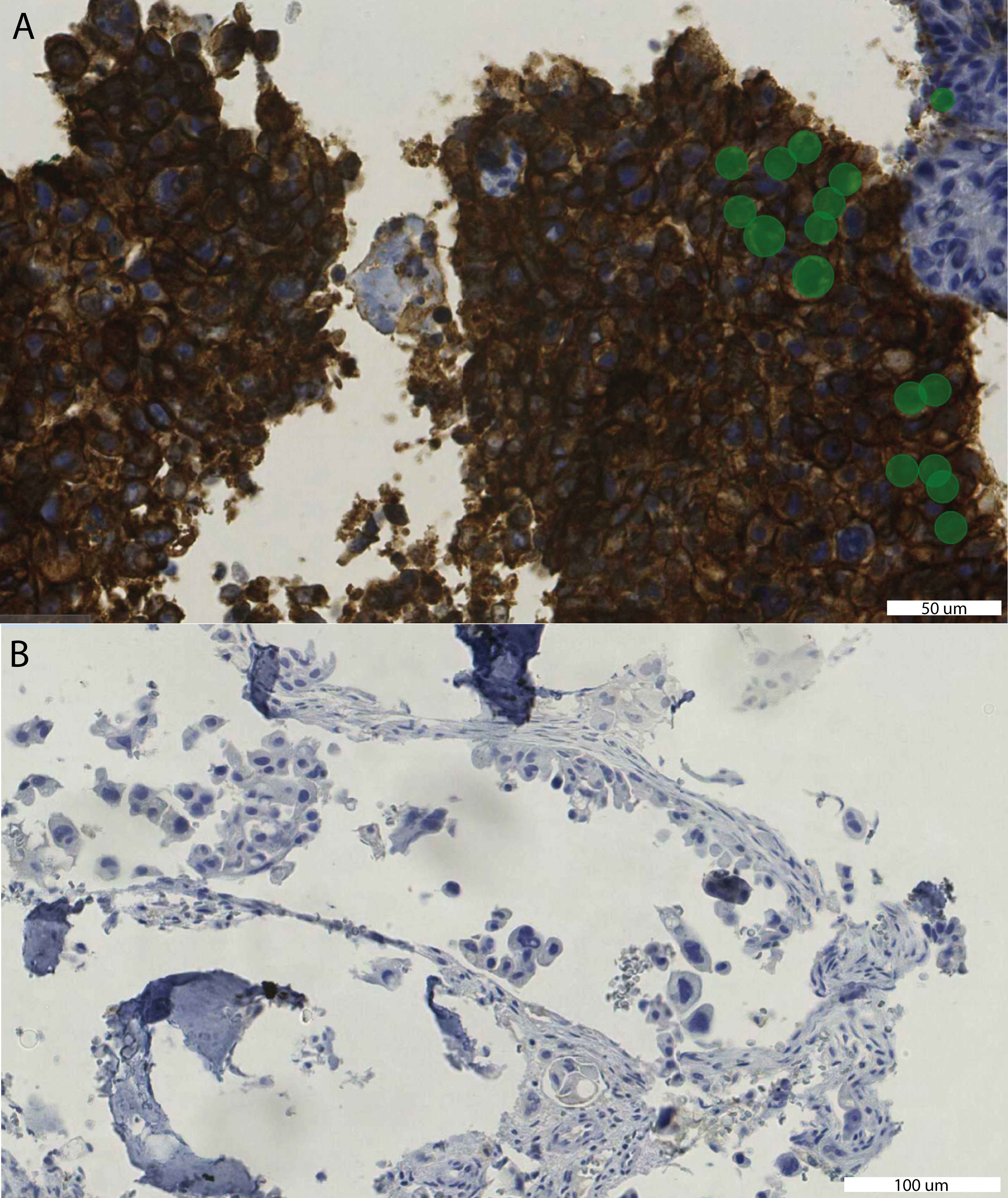

Supplement: Supplementary file 3 — Figure S3. Unscorable cases. [file HIS-80-635-s008.jpg]

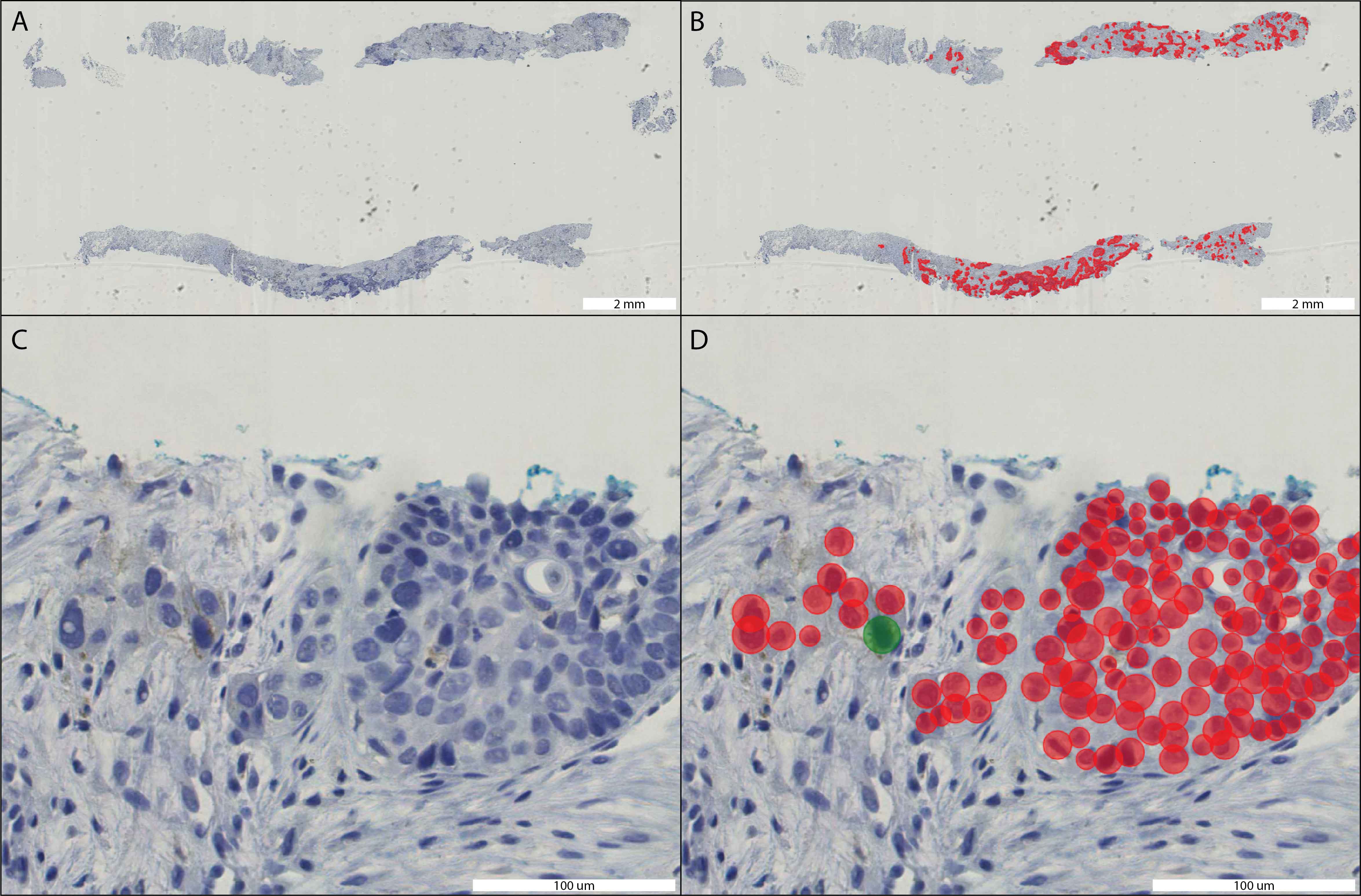

Supplement: Supplementary file 4 — Figure S4. Case example of algorithm scoring of a ‘difficult’ case between 0.5% and 60%. [file HIS-80-635-s003.jpg]

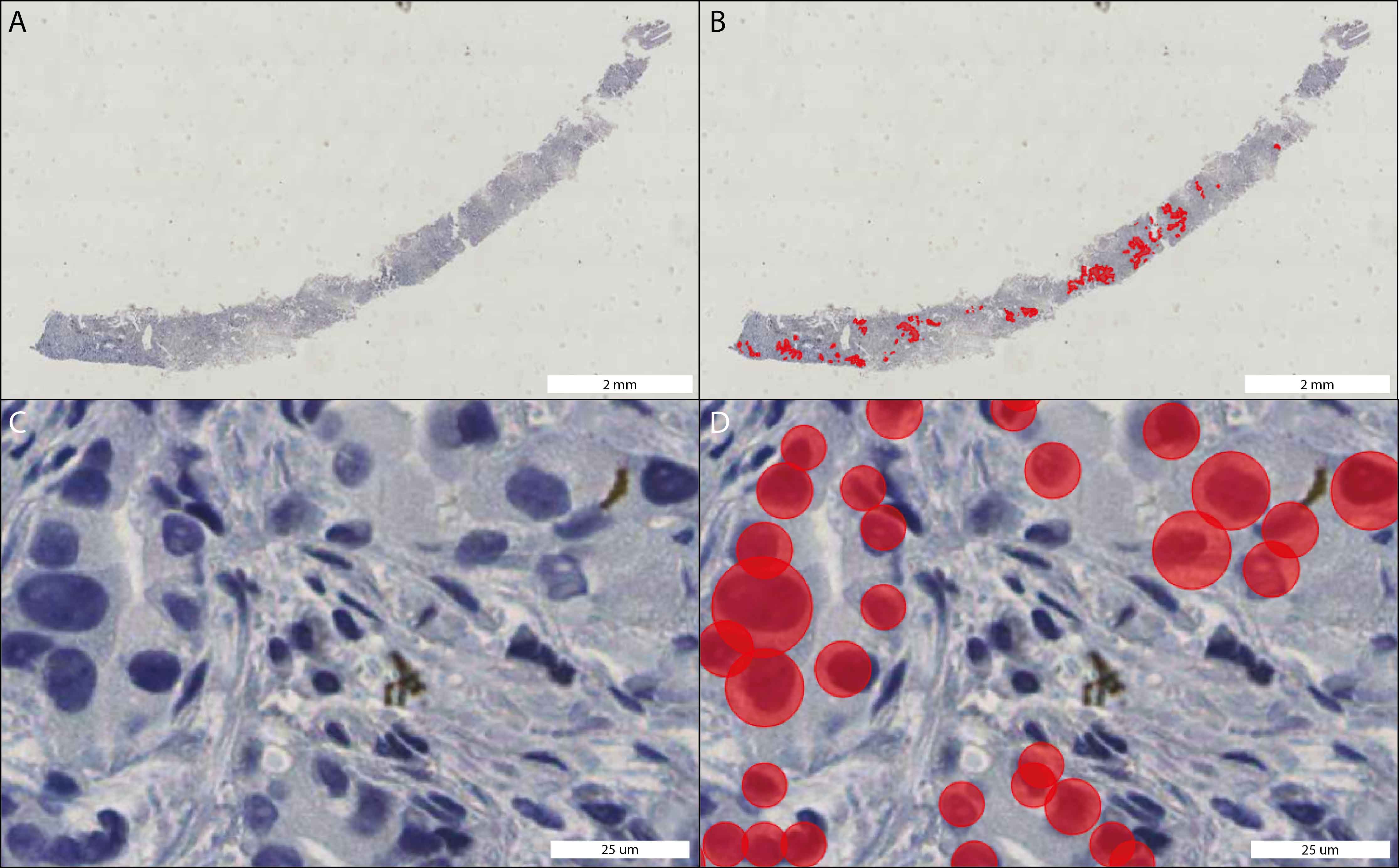

Supplement: Supplementary file 5 — Figure S5. Case example of algorithm scoring of an ‘easy’ case below 0.5%. [file HIS-80-635-s007.jpg]

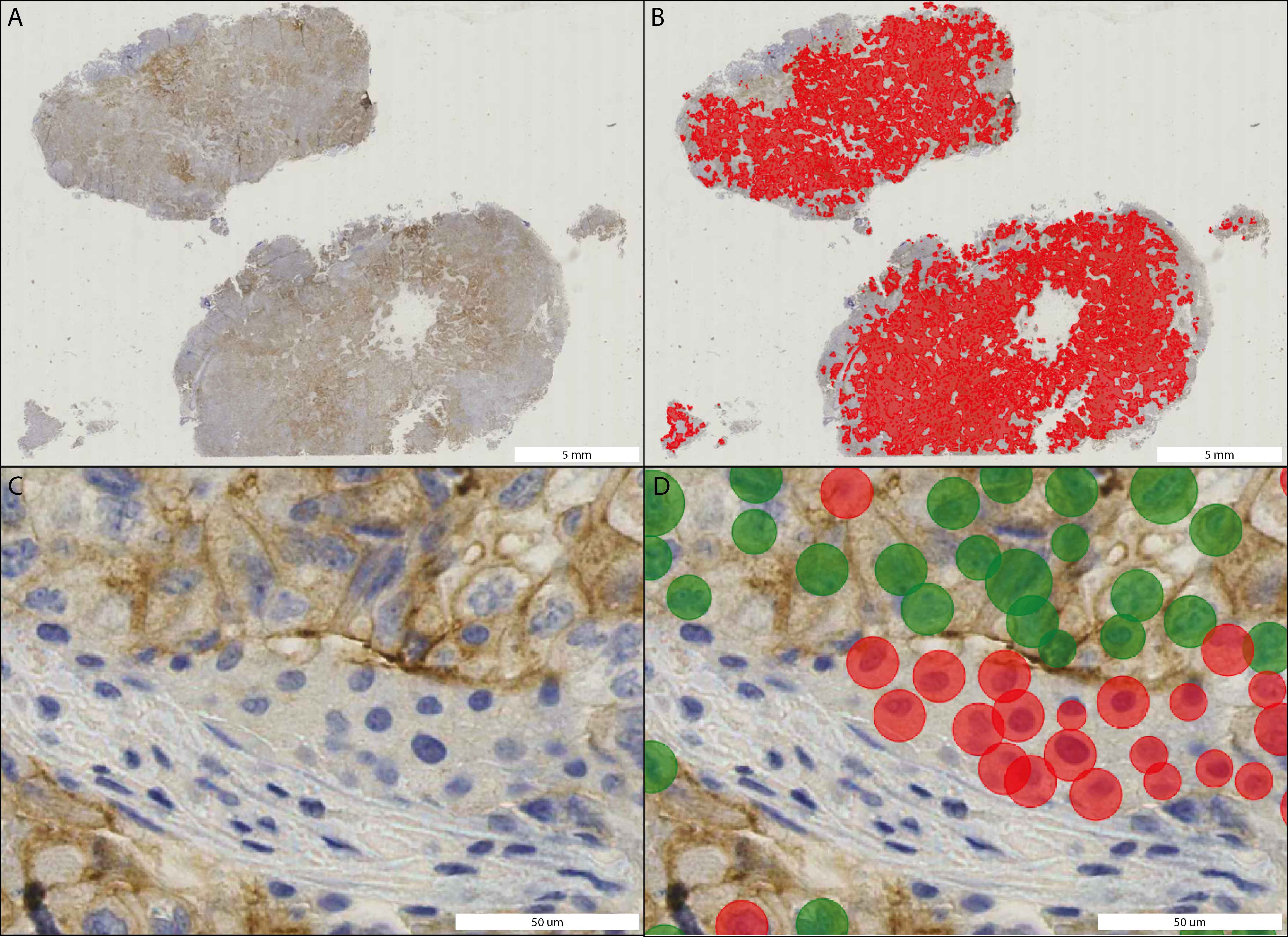

Supplement: Supplementary file 6 — Figure S6. Case example of algorithm scoring of an ‘easy’ case above 60%. [file HIS-80-635-s005.jpg]
