# Supplementary material for: Development and validation of a supervised deep learning algorithm for automated whole‐slide programmed death‐ligand 1 tumour proportion score assessment in non‐small cell lung cancer
Source: Histopathology. 2021 Nov 16;80(4):635–47. doi: 10.1111/his.14571 (PMC9299490; doi:10.1111/his.14571)
Supplement: Supplementary file 7 — Table S1. PD‐L1 algorithms in the literature and potential limitations. [file HIS-80-635-s001.docx]

**Supplementary Table**

|  | Widmaier | Kapil | Taylor | This study |
| --- | --- | --- | --- | --- |
| Case origin | Trial material | Trial material | Clinical retrospecitve | Clinical retrospective |
| Whole slide scoring | No | Yes | No, TMA | Yes |
| Number of observers (ground truth) | 1 | 1 | 3 | 3 |
| Manual annotation required | Yes | No | No | No |
| Microscopic feedback (explainable predictions) | Yes | No | Yes | Yes |
| Trained and validated on benign tissue areas | No | Yes | No | Yes |

*Supplementary Table 1: PD-L1 algorithms in the literature and potential limitations*
