# Supplementary material for: Development and validation of a supervised deep learning algorithm for automated whole‐slide programmed death‐ligand 1 tumour proportion score assessment in non‐small cell lung cancer
Source: Histopathology. 2021 Nov 16;80(4):635–47. doi: 10.1111/his.14571 (PMC9299490; doi:10.1111/his.14571)
Supplement: Supplementary file 8 — Data S1. Supplementary formulas. [file HIS-80-635-s004.docx]

**Supplementary Formulas**

$$TPS =\frac{PD-L1 positive tumor cells}{PD-L1 positive tumor cells+PD-L1 negative tumor cells}*100\%$$

*Supplementary Formula 1: tumor proportion score (TPS) is calculated via the formula above. TPS is used to measure PD-L1 expression in NSCLC specimens.*

$$Reference score=\frac{pathologist 1 TPS \left( \% \right)+pathologist 2 TPS \left( \% \right)+pathologist 3 TPS (\%)}{3}$$

*Supplementary Formula 2: calculation of the reference score by taking the mean of the three continuous pathologist scores (TPS).*
